# Differential Metabolic Responses to Adipose Atrophy Associated with Cancer Cachexia and Caloric Restriction in Rats and the Effect of Rikkunshito in Cancer Cachexia

**DOI:** 10.3390/ijms19123852

**Published:** 2018-12-03

**Authors:** Yuka Sudo, Hiroki Otsuka, Ryota Miyakawa, Akifumi Goto, Yohei Kashiwase, Kiyoshi Terawaki, Kanako Miyano, Yuto Hirao, Kanari Taki, Ryoma Tagawa, Masaki Kobayashi, Naoyuki Okita, Yasuhito Uezono, Yoshikazu Higami

**Affiliations:** 1 Laboratory of Molecular Pathology and Metabolic Disease, Faculty of Pharmaceutical Sciences, Tokyo University of Science, Chiba 278-8510, Japan; yukafeatkoro@me.com (Y.S.); hirohtsuka@yahoo.co.jp (H.O.); mykwryt.0518@gmail.com (R.M.); s2kaki@yahoo.co.jp (A.G.); yohei090109@yahoo.co.jp (Y.K.); 3B15077@ed.tus.ac.jp (Yu.H.); 3B18558@ed.tus.ac.jp (Ka.T.); tagawar@med.kobe-u.ac.jp (R.T.); kobayashim@rs.tus.ac.jp (M.K.); 2 Translational Research Center, Research Institute of Science and Technology, Tokyo University of Science, Chiba 278-8510, Japan; nokita7@rs.tusy.ac.jp (N.O.); yuezono@ncc.go.jp (Y.U.); 3 Division of Cancer Pathophysiology, National Cancer Research Institute, Tokyo 104-0045, Japan; terawaki_kiyoshi@mail.tsumura.co.jp (K.T.); kmiyano@ncc.go.jp (K.M.); 4 Tsumura Kampo Research Laboratories, Tsumura & Co., Ibaraki 300-1192, Japan; 5 Division of Supportive Care Research, National Cancer Center Exploratory Oncology Research and Clinical Trial Center, Tokyo 104-0045, Japan; 6 Division of Pathological Biochemistry, Faculty of Pharmaceutical Sciences, Sanyo-Onoda City University, Yamaguchi 756-0884, Japan

**Keywords:** cancer cachexia, caloric restriction, sterol regulatory element-binding transcription factor 1c, rikkunshito

## Abstract

Despite the similar phenotypes, including weight loss, reduction of food intake, and lower adiposity, associated with caloric restriction (CR) and cancer cachexia (CC), CC is a progressive wasting syndrome, while mild CR improves whole body metabolism. In the present study, we compared adipose metabolic changes in a novel rat model of CC, mild CR (70% of the food intake of control rats, which is similar to the food consumption of CC rats), and severe CR (30% of the food intake of controls). We show that CC and severe CR are associated with much smaller adipocytes with significantly lower mitochondrial DNA content; but, that mild CR is not. CC and both mild and severe CR similarly upregulated proteins involved in lipolysis. CC also downregulated proteins involved in fatty acid biosynthesis, but mild CR upregulated these. These findings suggest that CC might impair de novo fatty acid biosynthesis and reduce mitochondrial biogenesis, similar to severe CR. We also found that rikkunshito, a traditional Japanese herbal medicine, does not ameliorate the enhanced lipolysis and mitochondrial impairment, but rather, rescues de novo fatty acid biosynthesis, suggesting that rikkunshito administration might have partially similar effects to mild CR.

## 1. Introduction

Cancer cachexia (CC), a progressive wasting syndrome characterized by anorexia and weight loss, with depletion of both skeletal muscle and white adipose tissue (WAT), is observed in approximately 80% of patients with advanced cancer [1,2,3]. This syndrome causes serious clinical problems, which not only lead to poor quality of life (QOL) but also attenuate the effects of chemotherapy, and it is responsible for at least 20% of cancer-related mortality [4,5,6]. Pancreatic and stomach cancer patients, in particular, have a high risk of CC [7,8]. Both malabsorption and lower food intake contribute to weight loss in cancer patients, which is mainly the result of metabolic disturbances [9]. Recently, cachectic metabolic abnormalities have been shown to be in part due to substances released by tumors and pro-inflammatory cytokines [10,11,12,13].

The mechanisms of muscle atrophy in CC have been intensively studied and are accepted to involve a pathological decrease in protein synthesis coupled with an increase in protein degradation, mediated by both the ubiquitin-proteasome and lysosome systems [14,15]. In contrast, few studies have characterized the mechanisms of adipose tissue atrophy and the associated metabolic changes in CC. However, previous studies have shown that a higher rate of lipolysis is the major cause of adipose atrophy. For example, the turnover of glycerol and free fatty acid (FFA) was greater in cancer patients that were losing weight than in those who were not [16]. Thompson et al. [17] found that the expression of hormone-sensitive lipase (HSL), the major lipolytic enzyme, is higher in the adipocytes of cancer patients, resulting in higher serum triacylglycerol and FFA levels. Cytokines produced by tumors, such as interleukin 6 (IL-6) and tumor necrosis factor α (TNFα), have also been reported to be pro-lipolytic factors in CC. Moreover, in cachectic adipocytes, enhanced sensitivity to pro-lipolytic stimuli and greater production of pro-lipolytic factors have been demonstrated [18,19]. Recently, adipose triglyceride lipase (ATGL)-deficient mice have been shown to exhibit a resistance to CC, suggesting that lipolysis plays an essential role its pathogenesis [20]. However, the mechanisms of the adipose atrophy still remain incompletely understood.

WAT is a major tissue for energy storage and is now recognized as an endocrine organ that secretes biologically active molecules (adipokines). A recent study has shown that the quality of WAT and the adipokine profile affect whole-body metabolic homeostasis [21]. Long-term mild caloric restriction (CR) without malnutrition, (30% CR in our laboratory) reduces WAT mass (in other words WAT weight), reduces adipocyte size measured histologically, and improves the adipokine profile. CR is also well known to extend lifespan and delay the onset of age-associated disorders in a wide range of animal models [21,22,23,24]. We previously reported that the metabolic remodeling of WAT plays an important role in the anti-aging effect of 30% CR [25,26,27,28]. Recently, we have shown that 30% CR activates de novo fatty acid biosynthesis and mitochondrial biogenesis in WAT, and that the beneficial metabolic remodeling is due to transcriptional regulation by sterol regulatory element binding protein (SREBP-1c). Moreover, it is likely that SREBP-1c directly activates the peroxisome proliferator-activated receptor gamma coactivator 1-alpha (*Pgc1a*) promoter to induce CR-associated mitochondrial biogenesis [28]. Thirty percent CR and CC are associated with similar phenotypes, including weight loss, reduction in food intake, and lower adiposity. However, despite being characterized by similar food intake, CR improves whole body metabolism, whereas CC is associated with a deterioration in this.

Our group has previously established a novel rat model of severe cachexia and anorexia by implanting 85As2 cells derived from the MKN-45 human stomach cancer cell line into nude rats [29]. In this novel CC rat model, the plasma concentration of human leukemia inhibitory factor (LIF), an IL-6 family cytokine and known cachectic factor, is high. Moreover, the mRNA expression of hypothalamic orexigenic and anorexigenic peptides was upregulated and downregulated, respectively. Furthermore, we have shown that rikkunshito (RKT), a traditional Japanese herbal medicine, can ameliorate the weight loss and anorexia in this model of CC [29,30], and it has already been reported as ameliorating other types of anorexia [31,32,33]. Therefore, treatment with RKT may be beneficial for patients with anorexia-cachexia syndrome [34]. To our knowledge, however, no studies have described the effect of RKT on the metabolic changes associated with cachexic adipose tissue.

In the present study, we evaluated the adipose metabolic changes in our novel CC rat model by comparing the adipose metabolism associated with CC with that associated with a mild 30% CR (rats consuming 70% of the food intake of control rats, which is approximately equal food intake to that of CC rats) and with a severe 70% CR (rats consuming 30% of the food intake of control rats, which show an approximately similar reduction in adipose tissue mass to that of CC rats). We also investigated whether RKT could ameliorate the metabolic deterioration and anorexia. Then, we suggest that CC and severe CR might enhance lipolysis and over-activate mitochondria. We also suggest that CC might markedly suppress de novo fatty acid biosynthesis but mild CR significantly activate it probably transcriptionally activation of *Srebp-1*c. RKT is not able to ameliorate the abnormalities in lipolysis and mitochondrial function in CC. However, it is able to partially rescue the CC-associated impairment in de novo fatty acid biosynthesis, probably through a *Srebp-1c*-dependent mechanism.

## 2. Results

### 2.1. Fat and Muscle Mass

CC was associated with markedly lower food intake (70% of the food intake of control rats), and RKT slightly but significantly ameliorated this defect (Supplementary Figure S1A). The one-day food intake in CC rats (13.61 ± 1.80 g; 70.85% to food intake in control rats) to control rats (19.21 ± 1.30 g) was almost similar to that of 30% CR rats (Supplementary Figure S1A). CC was also characterized by significantly lower body mass, epididymal WAT (eWAT) mass, ratio of eWAT mass to body mass, *gastrocnemius* muscle mass, and *soleus* muscle mass; but RKT did not ameliorate these defects (Supplementary Figure S1B–F). In both 30% CR and 70% CR rats, body mass, eWAT mass, and ratio of eWAT mass to body mass were significantly lower than in rats fed *ad libitum* (AL) (Supplementary Figure S1G–I). The reduction rate of body weight and eWAT mass in CC rats to control rats was almost similar to that of 70% CR rats (Supplementary Figure S1B–D and S1G–I). In contrast to CC, both 30% and 70% CR did not affect *gastrocnemius* and *soleus* muscle masses (Supplementary Figure S1J,K).

### 2.2. Adipocyte Size

**Figure 1:**
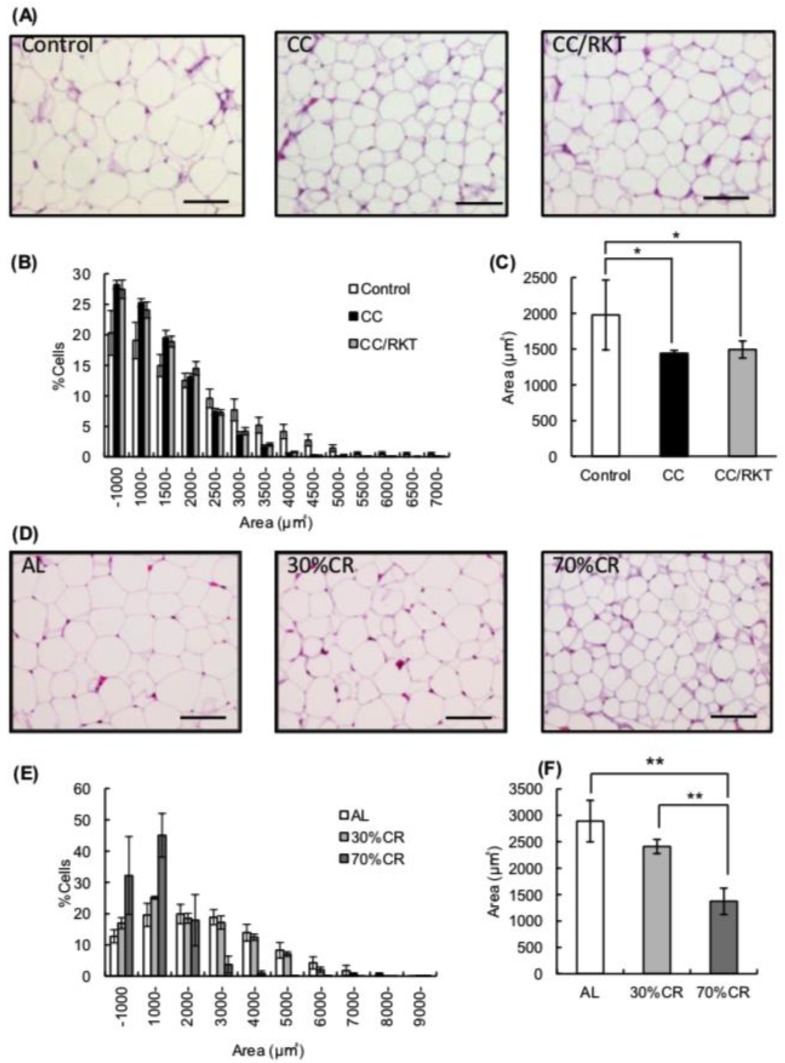
Effects of cancer cachexia (CC), rikkunshito (RKT) and caloric restriction (CR) on the histologic features of epididymal white adipose tissue (eWAT). Representative hematoxylin and eosin-stained histologic sections of eWAT from (**A**) control, CC, and CC/RKT rats and (**D**) rats fed ad libitum (AL) and subjected to CR (Magnification ×100, scale bar = 100 µm). Distribution of adipocyte size in (**B**) control, CC, and CC/RKT rats, and (**E**) AL and CR rats. The average of median adipocyte size in (**C**) control, CC, and CC/RKT rats, and (**F**) AL and CR rats. Error bars represent the SD associated with each mean (*n* = 5–6); * *p* < 0.05, ** *p* < 0.01 by Tukey’s test.

In CC and 70% CR rats, fat mass was markedly reduced and become less than half for two weeks as compared with control and AL rats, respectively. The reduction rate of the fat mass was more markedly in 70% CR rats than in CC rats (Supplementary Figure S1C,H). Next, we measured the size of adipocytes on histological sections. Rats with CC had much smaller white adipocytes (Figure 1A). The percentage of adipocytes of >3000 µm^2^ was lower (Figure 1B) and the median adipocyte size in CC rats (1440 ± 43 µm^2^) was significantly lower than in controls (1977 ± 489 µm^2^) (Figure 1C). RKT did not affect adipocyte size (Figure 1A–C). In contrast, 30% CR rats had slightly smaller adipocytes than controls, but this difference was not significant. Seventy percent of the CR rats had much smaller adipocyte sizes and significantly fewer large adipocytes (>3000 µm^2^) (Figure 1D,E). The median adipocyte size in 30% CR rats (2409 ± 135 µm^2^) was not significantly lower than in AL rats (2899 ± 394 µm^2^), but that in 70% CR rats (1372 ± 250 µm^2^) was markedly lower than in both AL and 30% CR rats (Figure 1F). When we compared the reduction rate between fat mass and median adipocyte size by CC with that by CR, it appeared that CC significantly reduces number of adipocyte in WAT as compared with 30% and 70% CR. In addition, we did not find any browning or baizing change of adipocytes in WAT of CC, 30% CR and 70% CR rats histologically.

### 2.3. Expression Levels of Proteins Involved in Lipid Metabolism

**Figure 2:**
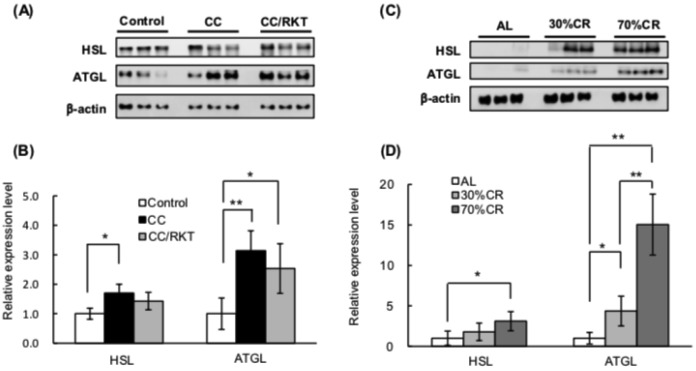
Effects of cancer cachexia (CC), rikkunshito (RKT), and caloric restriction (CR) on the protein expression of key lipases in epididymal white adipose tissue (eWAT). Western blot analysis of hormone-sensitive lipase (HSL) and adipocyte triglyceride lipase (ATGL) was performed using a chemiluminescence method. Lysates were prepared from eWAT of control, CC, and CC/RKT rats (**A**,**B**), rats fed ad libitum (AL) and subjected to CR (**C**,**D**). (**A**,**C**) Representative images of western blots. (**B**,**D**) Densitometry data for HSL and ATGL. The expression of each protein was normalized to β-actin protein expression. Error bars represent the SD associated with each mean (*n* = 5–6). * *p* < 0.05, ** *p* < 0.01 by Tukey’s test.

To compare lipid metabolism in CC and CR rats, we measured the expression levels of the key lipases, hormone sensitive lipase (HSL) and adipocyte triglyceride lipase (ATGL), in WAT. CC was associated with significantly higher protein levels of HSL and ATGL. RKT slightly ameliorated the CC-associated upregulation of HSL, but not significantly (Figure 2A,B). Thirty percent CR significantly increased the expression of ATGL but not HSL, whereas 70% CR markedly increased the expression of both enzymes (Figure 2C,D).

**Figure 3:**
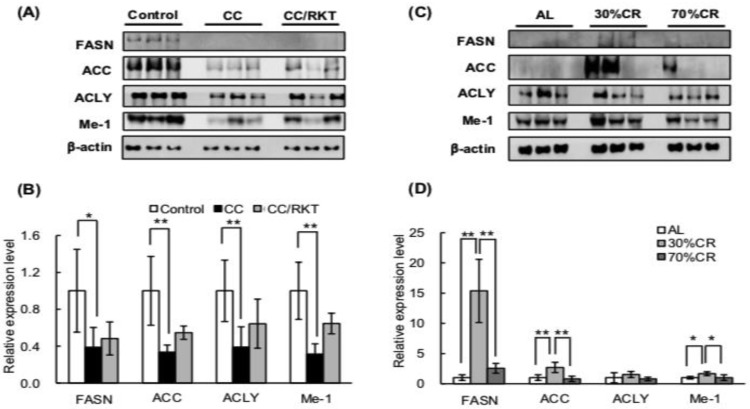
Effects of cancer cachexia (CC), rikkunshito (RKT), and caloric restriction (CR) on the expression of proteins involved in de novo fatty acid biosynthesis in epididymal white adipose tissue (eWAT). Western blot analysis of fatty acid synthase (FASN), acetyl-CoA carboxylase (ACC), ATP citrate lyase (ACLY), and malic enzyme 1 (Me-1) was performed using a chemiluminescence method. Protein samples were extracted from eWAT of (**A**,**B**) control, CC, and CC/RKT rats, and (**C**,**D**) rats fed ad libitum (AL) and subjected to CR. (**A**,**C**) Representative images of western blots. (**B**,**D**) Densitometry data for FASN, ACC, ACLY, and Me-1. Protein levels were normalized to β-actin protein expression. Error bars represent the SD associated with each mean (*n* = 5–6). * *p* < 0.05, ** *p* < 0.01 by Tukey’s test.

We next measured the expression levels of proteins involved in fatty acid biosynthesis: fatty acid synthase (FASN), acetyl-CoA carboxylase (ACC) and ATP citrate lyase (ACLY). Malic enzyme 1 (Me-1) catalyzes oxidative decarboxyration of malate to pyruvate in cytoplasm. Importantly, Me-1 generates NADPH, which is a critical substrate for fatty acid biosynthesis [35]. We therefore examined the expression of Me-1 as well. CC was associated with markedly lower expression of all of these proteins (Figure 3A,B). RKT ameliorated this CC-associated downregulation slightly. Resultantly, the expression level of these proteins in CC/RKT was not significantly lower than that in control. In contrast, 30% CR significantly upregulated the expression of FASN, ACC, and Me-1, but 70% CR did not affect the expression of these proteins (Figure 3C,D).

**Figure 4:**
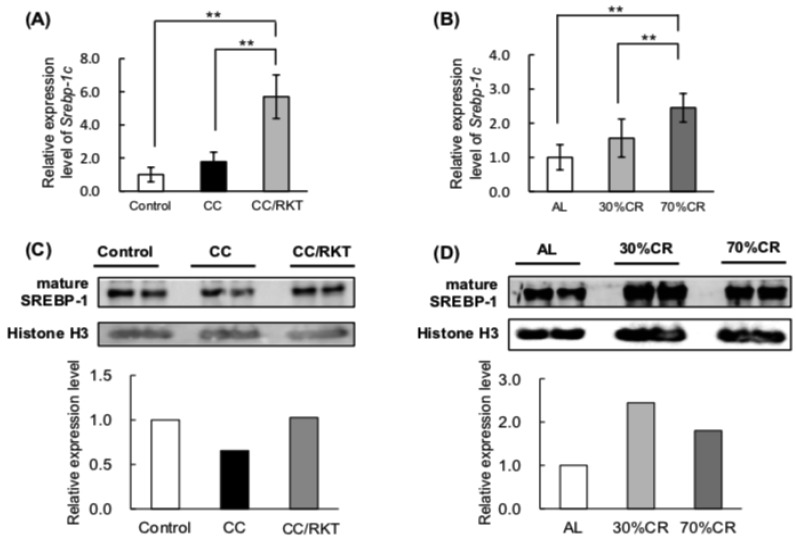
Effects of cancer cachexia (CC), rikkunshito (RKT), and caloric restriction (CR) on the expression of *Srebp-1c* mRNA and the mature active form of SREBP-1 protein in epididymal white adipose tissue (eWAT). To measure *Srebp-1*c mRNA by real-time RT-PCR, total RNA was extracted from the eWAT of (**A**) control, CC, and CC/RKT rats, and (**B**) rats fed ad libitum (AL) and subjected to CR. *Srebp-1*c mRNA levels were normalized to *Tbp* mRNA expression. Error bars represent the SD associated with each mean (*n* = 5–6). ** *p* < 0.01 by Tukey’s test. Western blot analysis of SREBP-1 was performed using a chemiluminescence method. (**C**,**D**) Image of a western blot for the mature form of SREBP-1. Pooled nuclear extracts from the eWAT of three (**C**) control, CC, or CC/RKT rats, and (**D**) AL or CR rats were used in each lane, resultantly pooled nuclear extracts from total six rats were analyzed. Densitometry data for SREBP-1 protein were normalized to data for histone H3 protein.

Because SREBP-1c transcriptionally regulates the expression of proteins involved in fatty acid biosynthesis [36], we also measured the mRNA expression of *Srebp-1c*. Neither CC nor 30% CR rats showed differences in expression of *Srebp-1c* mRNA from controls, but RKT markedly upregulated this in CC rats, as did 70% CR (Figure 4A,B). SREBP-1c is synthesized as a long inactive precursor, which is then cleaved to yield the active mature form, which can then enter the nucleus to activate transcription [37]. We therefore investigated whether SREBP-1c transcriptionally mediates the CC-, RKT-, and CR-associated differences in the expression of proteins involved in fatty acid biosynthesis. Unfortunately, to our knowledge, there are no antibodies available that target SREBP-1c. Therefore, we measured the active mature form of SREBP-1 protein in nuclear extracts prepared from pooled WAT samples by western blotting, using an antibody against SREBP-1. Mature SREBP-1 protein expression was lower in CC rats, but treatment with RKT ameliorated this defect (Figure 4C). Both 30% and 70% CR increased the expression of the active mature form of SREBP-1 protein, but the increase was more pronounced in 30% CR than 70% CR rats (Figure 4D).

### 2.4. Expression and Activity of Mitochondrial Factors

**Figure 5:**
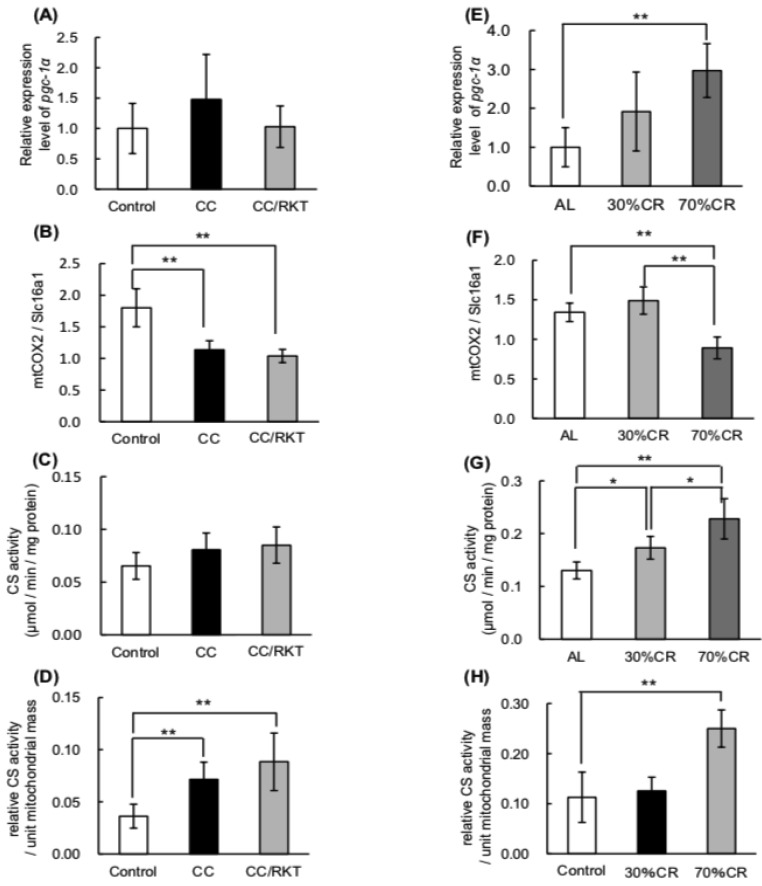
Effects of cancer cachexia (CC), rikkunshito (RKT), and caloric restriction (CR) on the expression of *Pgc-1α* mRNA, mitochondrial DNA (mtDNA) content, and citrate synthase (CS) activity in epididymal white adipose tissue (eWAT). To measure *Pgc-1α* mRNA levels by real-time RT-PCR, total RNA was extracted from eWAT of (**A**) control, CC, and CC/RKT rats, and (**E**) rats fed ad libitum (AL) and subjected to CR. *Pgc-1α* mRNA levels were normalized to *Tbp* mRNA levels. The ratio of mitochondrial (COX2) to nuclear (SLC16A1) DNA was obtained using real-time PCR and DNA extracted from eWAT of (**B**) control, CC, and CC/RKT rats, and (**F**) AL and CR rats. Ratios are expressed as fold changes relative to mean values for control or AL rats. Citrate synthase (CS) activity in eWAT of (**C**) control, CC, and CC/RKT rats, and (**G**) AL and CR rats, was measured spectrophotometrically at 412 nm. CS activity to mtDNA content ratio, indicative of mitochondrial enzymatic activity per unit mitochondrial mass, was calculated for the eWAT of (**D**) control, CC, and CC/RKT rats, and (**H**) AL and CR rats. Error bars represent the SD associated with each mean (*n* = 5–6). * *p* < 0.05, ** *p* < 0.01 by Tukey’s test.

We have previously reported that 30% CR activates mitochondrial biogenesis in an *Srebp-1c*-dependent manner. Moreover, SREBP-1c directly binds and activates the *Pgc-1α* promoter to induce CR-associated mitochondrial biogenesis [28]. Therefore, we investigated the effects of CC and CR on mitochondrial function. CC and RKT did not affect significantly the expression of *Pgc-1α* mRNA (Figure 5A). Seventy percent CR significantly upregulated the expression of *Pgc-1α* mRNA (Figure 5E). In contrast, CC was associated with lower mitochondrial (mt)DNA content, determined by calculating a ratio of cytochrome c oxidase subunit 2 (COX2) DNA (mitochondrial) to solute carrier family 16, member 1 (Slc16a1) DNA (nuclear), but RKT did not affect this parameter (Figure 5B). Thirty percent CR did not affect mtDNA content, but 70% CR significantly reduced this (Figure 5F). Whereas CC and RKT did not affect citrate synthase (CS) activity (Figure 5C), 30% and 70% CR increased CS activity, and the degree of activation was greater in 70% CR than 30% CR rats (Figure 5G). Finally, we calculated the CS activity to mtDNA content ratio, indicative of mitochondrial enzymatic activity per unit mitochondrial mass. CC markedly increased mitochondrial enzymatic activity per unit mitochondrial mass, but RKT did not ameliorate this defect (Figure 5D). Thirty percent of the CR did not affect mitochondrial enzymatic activity per unit mitochondrial mass, but 70% CR significantly increased this index (Figure 5H).

## 3. Discussion

The severity of body fat loss in cancer patients correlate with mortality rate [38,39,40]. It is well accepted that a greater lipolytic rate is a common defect in cachexia patients and various experimental CC models [19,41,42,43,44]. Das et al. [20] reported that a greater lipolytic rate is essential for the pathogenesis of CC, including for myocyte apoptosis and proteasomal degradation. In particular, they clearly showed that ATGL is more important for CC-associated pathology than HSL, using both ATGL- and HSL-deficient mice. Our novel CC rat model is characterized by low WAT mass and small adipocytes, which are accompanied by significantly higher expression of both HSL and ATGL, but the difference in expression of ATGL from that of control rats is more marked than for HSL. These findings are consistent with the previous report by Das et al. [20] that showed that both 70% and 30% CR reduced WAT mass, in the presence and absence of a significant reduction in adipocyte size, respectively. Moreover, 70% CR markedly upregulated both HSL and ATGL expression, and the upregulation of ATGL was greater than that of HSL, suggesting that CC is associated with a similar metabolic effect on lipolysis to that of 70% CR.

CC was associated with lower expression of the active mature form of SREBP-1 protein, but did not affect *Srebp-1*c mRNA level. Moreover, transcriptional target proteins of SREBP-1c, which are involved in de novo fatty acid biosynthesis, were also expressed at much lower levels in CC rats. In contrast, and in spite of their similar food intake to that of CC rats, 30% CR increased the expression of these proteins. Bing et al. [45] reported that the expression of *Srebp-1c* mRNA and SREBP-1 protein, and its target lipogenic genes, including *Fasn* and *Acc*, was significantly lower in a mouse model of CC created by the implantation of MAC16 colon adenocarcinoma cells. However, despite the use of different CC models, lower expression of the active mature form of SREBP-1 protein and its target lipogenic gene expression in WAT was consistently observed. Using *Srebp-1c*-deficient mice, we have also shown that SREBP-1c is a key mediator of the beneficial metabolic effects, including the enhancement in de novo fatty acid biosynthesis, in the WAT of CR mice [28]. Moreover, we observed that 70% CR for 2 weeks did not downregulate these proteins. Therefore, it seems that defective de novo fatty acid biosynthesis in WAT is a common pathology in CC models, but that it is not responsible for the lower food intake. We have previously shown that plasma LIF levels are much higher in CC rats [29]. LIF is a tumor-derived factor that is involved in the development of cachexia/anorexia, alongside IL-6 and TNFα [11,12,13]. LIF suppresses lipid storage by inhibiting lipoprotein lipase in WAT [46], and although it has a limited effect on lipolysis in 3T3-L1 adipocytes [47], it significantly downregulates the expression of proteins involved in fatty acid biosynthesis, including SREBP-1 and FASN, in this cell line [48]. We therefore consider that 85As2-derived LIF might be one of the factors involved in the suppression of fatty acid biosynthesis in our CC model.

In our CC rat model, the expression of *Pgc-1α* mRNA was slightly but not significantly higher than in controls. In contrast, in mice in which CC was induced using MAC16 colon adenocarcinoma cells, it was much higher [45]. This difference between the CC models might be due to the different species used. However, CC and 70% CR had similar effects on mitochondrial enzymatic activity per unit mitochondrial mass, suggesting that both phenotypes may involve the wasteful consumption of energy by excessive mitochondrial activity.

We have reported previously that RKT ameliorates several of the defects associated with cancer cachexia in our novel CC rat model [29,30]. We show here that RKT did not affect lipolysis or mitochondrial function. However, RKT treatment significantly upregulated *Srebp-1c* mRNA and the active mature form of SREBP-1 protein, and partly suppressed the CC-associated reduction of protein expressions involved in fatty acid biosynthesis, suggesting that RKT might partly restore de novo fatty acid biosynthesis in WAT. Because refeeding after fasting upregulates the expression of SREBP-1c, it is possible that effect of RKT on SREBP-1c was the result of an RKT-induced increase in food intake. It is widely accepted that the beneficial effect of RKT on anorexia is exerted through the activation of signaling by the appetite-stimulating peptide ghrelin [49]. RKT increases both serum ghrelin [50] and ghrelin receptor sensitivity, in part by activating phosphodiesterase 3B [51], and ghrelin signaling modulates not only hypothalamic, but also peripheral, lipid metabolism [52,53]. It has been reported that ghrelin administration increases the expression of proteins involved in fatty acid biosynthesis in WAT in a p53-dependent manner [54]. Therefore, we hypothesize that the beneficial metabolic effects of RKT might be exerted in WAT through a ghrelin-dependent mechanism, rather than through an effect on food intake.

It is widely accepted that small adipocytes, which secrete more adiponectin and less inflammatory adipokines, are beneficial for whole body metabolism [21]. On the basis of a comparison between CC and CR rats, we suggest that CC reduces and miniaturizes adipocytes, enhances lipolysis, suppresses de novo fatty acid biosynthesis and over-activates mitochondria in WAT. In contrast to 30% CR, it is likely that the small adipocytes observed in CC rats are not beneficial for whole body metabolism. We have previously suggested that 30% CR-associated metabolic remodeling, including greater de novo fatty acid biosynthesis and mitochondrial biogenesis in WAT, are involved in making energy use more efficient in the face of insufficient energy intake [25,28]. We show here that this energy-saving system, which means that less energy can be used more efficiently in WAT, is partly impaired in CC. We also show that RKT is not able to ameliorate the abnormalities in lipolysis and mitochondrial function in CC. However, it might partially rescue the CC-associated impairment in de novo fatty acid biosynthesis, probably through a *Srebp-1c*-dependent mechanism. To clarify the molecular mechanism of the effect of RKT on fatty acid biosynthesis, further studies, particularly regarding whether RKT has its effects through peripheral ghrelin signaling, are required. At present, however, RKT is widely prescribed to the patients with various upper gastrointestinal symptoms including anorexia, dyspepsia and vomiting. Hence, we also want to emphasize that RKT may be a promising medicine for the CC patients.

## 4. Materials and Methods

### 4.1. Animal Experiments

The present study was conducted in accordance with provisions of the Ethics Review Committee for Animal Experimentation at the National Cancer Research Institute of Japan (Approval nos. T09-050-M02 (January 6, 2011), T09-050-C04 (April 2, 2012) and T17-045 (May 17, 2017)) and Tokyo University of Science (Approval nos. Y16049 (June 1, 2016) and Y17051 (June 8, 2017)). Cachexia model rats were established as previously reported [29]. Briefly, male nude rats (F344/NJcl-mu/mu) were purchased from Clea-Japan Inc. (Tokyo, Japan) at 6 weeks of age. At 8 weeks of age, rats were implanted subcutaneously with either human gastric carcinoma 85As2 cells or phosphate buffered saline (as control). Rikkunshito (RKT, Tsumura & Co., Tokyo, Japan) was prepared and administered as previously described [29,30]. Tumor-bearing CC rats were divided into two groups at 10 weeks of age: a control group (CC), which was administered distilled water (10 mL/kg), and a treatment group (CC/RKT), which was administered RKT orally twice daily at 1000 mg·kg^-1^·day^-1^ for 7 days. At 11 weeks of age the rats were euthanized using isoflurane overdose (Mylan, Canonsburg, PA, USA). During the experimental period, any tumor-bearing CC rats were not died.

Male Wistar rats aged 5 weeks were purchased from Clea-Japan Inc. and were maintained under Specific Pathogen-Free conditions at 23 °C, under a 12 h light-dark cycle, in the Laboratory Animal Center at the Faculty of Pharmaceutical Sciences, Tokyo University of Science. The rats were provided with water and fed *ad libitum* with a Labo MR Stock diet (NOSAN, Yokohama, Japan). At 12 weeks of age, rats were divided into three groups: one was fed *ad libitum* (AL) and the others were calorie-restricted (to either 30% or 70% of the AL energy intake). The one-day food intake was 20.37 ± 0.89 g in AL rats. 70% and 30% food intake of AL rats was provided in 30% and 70% CR rats, respectively. 70% CR at least for 2 weeks is not a lethal dietary intervention. At 14 weeks of age, all rats were euthanized under anesthesia by isoflurane inhalation 3–5 h after turning on the lights, and eWAT was collected and weighed. WAT samples were immediately diced, frozen in liquid nitrogen, and stored at −80 °C. Part of the isolated WAT was fixed in buffered formalin solution, processed routinely, and embedded in paraffin for histologic examination.

### 4.2. Histologic Examination

Fixed tissues from rats were sectioned at 5 µm and stained with hematoxylin and eosin (HE). Stained sections were assessed by microscopy using a CCD camera (Nikon, Tokyo, Japan). The size distributions for each white area in the black-and-white images, indicative of lipid droplets, were measured and calculated using ImageJ 1.43u/Java1.6.0_22 software (National Institute of Mental Health, Bethesda, Maryland, USA).

### 4.3. Protein Extraction and Western Blot Analysis of Target Protein Levels

Preparation of WAT lysates, nuclear protein extraction, and western blotting were performed as described previously [25,27]. Equal amounts of protein (5–20 mg) were subjected to SDS–PAGE and transferred to nitrocellulose membranes. After blocking with skim milk and BSA, the membranes were probed with the appropriate primary antibodies. After washing, membranes were incubated with the appropriate secondary antibody, and subsequently incubated with ImmunoStar LD reagent (Wako) after further washing. Specific proteins were visualized using LAS3000 (Fujifilm, Tokyo, Japan), and the data were analyzed using Multigauge software (Fujifilm, Version3.1, Tokyo, Japan). The following primary antibodies were utilized: β-Actin (A1978, Sigma-Aldrich, Saint Louis, MO, USA), FASN (616092, BD Transduction Laboratories, San Joes, CA, USA), ACC (no. 3662, Cell Signaling Technology Inc., Danvers, MA, USA), ATGL (no. 2439, Cell Signaling Technology Inc.), ACLY (1822-1, Epitomics), HSL (no. 4107, Cell Signaling Technology Inc.), ME-1 (SAB4501853, Sigma-Aldrich), SREBP-1 (sc-8984, Santa Cruz, Dallas, TX, USA) and histone H3 (no. 4499, Cell Signaling). Secondary antibodies were purchased from Jackson Immunological Research Laboratories (West Grove, PA, USA): horseradish peroxidase-conjugated F(ab’)2 fragment of goat anti-mouse IgG (115-036-062) and horseradish peroxidase-conjugated F(ab’)2 fragment of goat anti-rabbit IgG (115-036-045).

### 4.4. RNA Extraction and Real-Time Reverse-Transcription Polymerase Chain Reaction

Total RNA was extracted from frozen WAT using RNAiso PLUS (Takara, Shiga, Japan) and purified using the FastPure RNA kit (Takara), according to the manufacturer’s protocol. To obtain cDNA, 1 µg RNA was subjected to reverse transcription using PrimeScript Reverse Transcriptase (Takara), with random hexamer primers (Takara). Real-time quantitative PCR was performed using the Applied Biosystems 7300 Real-time PCR system (Life Technologies, Grand Island, NY,USA) and SYBR Premix ExTaqII (Takara), as reported previously [25]. *Srebp-1c*, *Pgc-1α* and TATA box binding protein *(Tbp)* transcripts were amplified; *Tbp* was used as a reference gene. The following primers were used: *Srebp-1c*: 5′-GGA GCC ATG GAT TGC ACA TT-3′ (forward) and 5′-GGC CCG GGA AGT CAC TGT-3′ (reverse), *Pgc-1α*: 5′-AGA CGG ATT GCC CTC ATT TG-3′ (forward) and 5′-CAG GGT TTG TTC TGA TCC TGT G-3′ (reverse), *Tbp*: 5′-CCC TCA CAC TCA GAT CAT CTT CTC-3′ (forward) and 5′-GCC TTG TCC CTT GAA GAG AAC C-3′ (reverse).

### 4.5. Analysis of Citrate Synthase Activity

The activity of citrate synthase (CS) was measured as previously reported, with some modifications [55]. Briefly, WAT samples were homogenized in homogenization buffer containing 50 mM Tris-HCl, pH 7.4, 150 mM NaCl, 1% phosphatase inhibitor cocktail, 5 mM EDTA, 1% protease inhibitor cocktail, 1% Triton X-100, and 0.05% sodium deoxycholate. Protein concentration was determined using a BCA protein assay kit (Thermo Scientific, Rockford, IL, USA), according to the manufacturer’s protocol. For CS activity measurements, a reaction mixture containing 0.1 mM 5,5-dithiobis-(2-nitrobenzoic) acid (Wako), 0.5 mM acetyl-CoA (Wako), 0.1% Triton X-100, and 100 mM Tris-HCl, pH 8.0, was used with the tissue homogenates, which contained 5–8 mg protein. After incubation at 25 °C for 5 min, the absorbance at 412 nm (SpectraMax Plus384, Molecular Devices, Sunnyvale, CA, USA) was measured over 3 min to determine the non-specific activity. Reactions were then initiated by addition of 0.5 mM oxaloacetate (Wako) in a final volume of 200 mL, and the change in absorbance was recorded for at least 3 min.

### 4.6. Analysis of mtDNA Content

mtDNA was extracted from WAT by digestion with proteinase K and 10% SDS (100 mg/mL) in a buffer containing 150 mM NaCl, 10 mM Tris-HCl, pH 8.0, and 10 mM EDTA. Homogenate samples were incubated overnight at 55 °C, then an equal volume of phenol was added, and the samples were rotated for 1 h. After centrifugation, the aqueous phase was removed to a fresh tube and rotated with an equal volume of PCI (phenol/chloroform/isoamyl alcohol) for 1 h. To remove RNA, the aqueous phase was incubated with 0.02 volume of RNase A (Wako, Osaka, Japan) for 1 h at 37 °C. These phenol and PCI extractions were then repeated. DNA was precipitated from the aqueous phase by adding 0.1 volume of 3 M sodium acetate after adding two volumes of 100% ethanol. The DNA was washed twice with 180 mL of 70% ethanol, gently air-dried, and re-suspended in tris-EDTA buffer. The concentration was adjusted to 20 ng/mL.

A quantitative PCR assay was performed using the Applied Biosystems 7300 Real-time PCR system (Life Technologies, Carlsbad, CA, USA) [56]. mtDNA was amplified by real-time PCR for SLC16A1 and COX2. The primer sequences for real-time PCR analysis of SLC16A1 and COX2 were: SLC16A1: 5′-TAG CTG GAT CCC TGA TGC GA-3′ (forward), 5′-GCA TCA GAC TTC CCA GCT TCC-3′ (reverse); COX2: 5′-CTT ACA AGA CGC CAC ATC AC-3′ (forward), 5′-GAA TTC GTA GGG AGG GAA GG-3′ (reverse) [57]. The relative amounts of mtDNA were expressed as ratios of COX2 DNA/SLC16A1 DNA.

### 4.7. Statistical Analysis

All data were derived from five to six rats per group, are expressed as means ± SDs, and were analyzed using Tukey’s test. Differences with *p* < 0.05 were considered to be statistically significant.

## Supplementary Materials

Supplementary materials can be found at http://www.mdpi.com/1422-0067/19/12/3852/s1.
