# Supplementary material for: Differential Metabolic Responses to Adipose Atrophy Associated with Cancer Cachexia and Caloric Restriction in Rats and the Effect of Rikkunshito in Cancer Cachexia
Source: Int J Mol Sci. 2018 Dec 3;19(12):3852. doi: 10.3390/ijms19123852 (PMC6321026; doi:10.3390/ijms19123852)
Supplement: Supplementary file 1 [file ijms-19-03852-s001.pdf]

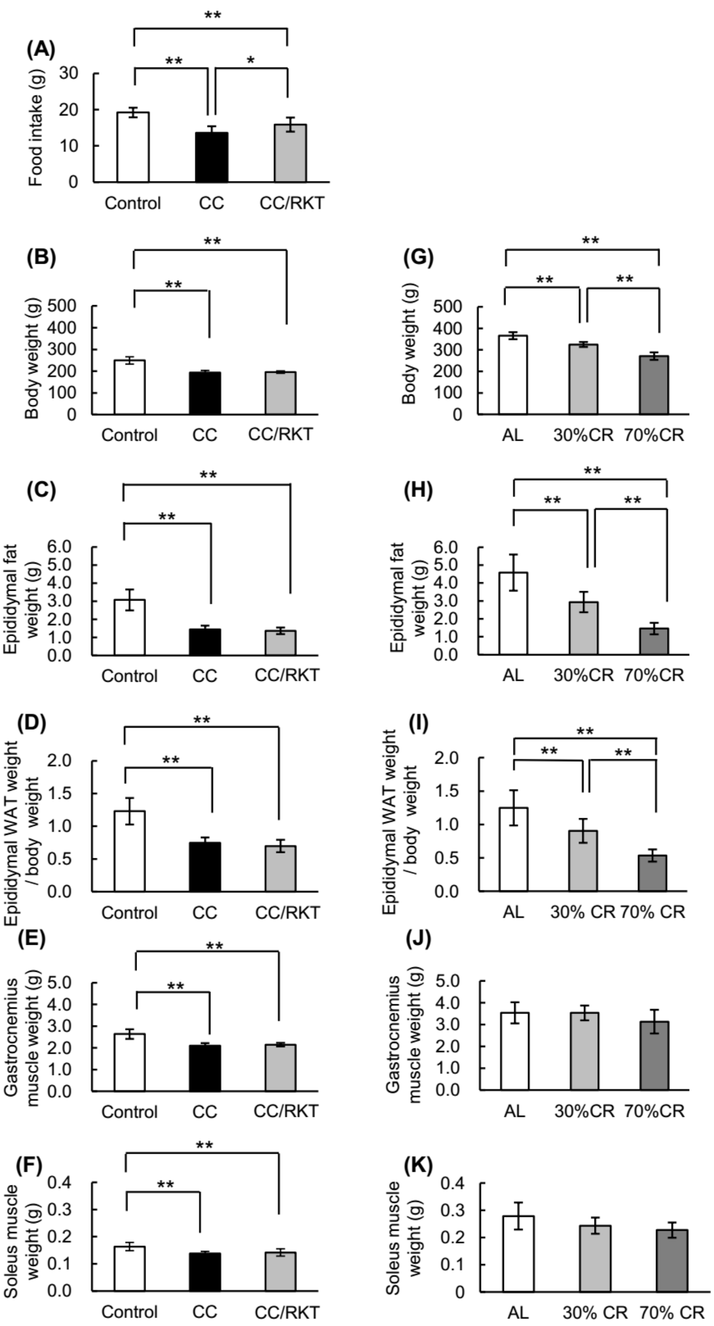

Supplementary Figure S1. Effects of cancer cachexia (CC), rikkunshito (RKT), and caloric restriction (CR) on the food intake, body weight, epididymal white adipose tissue (eWAT) weight and muscle weight. (**A**) Food intake of control and CC rats. (**B**,**G**) Body weight of CC and CC/RKT rats (**B**), and AL and CR rats (**G**). (**C**,**H**) Epididymal fat weight of CC and CC/RKT rats (**C**), and (**H**) AL and CR rats (**H**). (**D**,**F**) Epididymal fat weight per body weight of CC and CC/RKT rats (**D**), and AL and CR rats (**I**). (**E**,**J**) Gastrocunemius muscle weight of CC and CC/RKT rats (**E**), and AL and CR rats (**J**). (**F**,**K**) Soleus muscle weight of CC and CC/RKT rats (**F**), and AL and CR rats (**K**). Error bars represent the SD associated with each mean (n = 5–6). * $p < 0.05$, ** $p < 0.01$ by Tukey's test.
